# Two-Dimensional Bimetallic Phthalocyanine Covalent-Organic-Framework-Based Chemiresistive Gas Sensor for ppb-Level NO_2_ Detection

**DOI:** 10.3390/nano13101660

**Published:** 2023-05-17

**Authors:** Xiyu Chen, Min Zeng, Jianhua Yang, Nantao Hu, Xiaoyong Duan, Wei Cai, Yanjie Su, Zhi Yang

**Affiliations:** 1 Key Laboratory of Thin Film and Microfabrication (Ministry of Education), Department of Micro/Nano Electronics, School of Electronic Information and Electrical Engineering, Shanghai Jiao Tong University, Shanghai 200240, China; chenxiao00yu@sjtu.edu.cn (X.C.); yangjh08@sjtu.edu.cn (J.Y.); yanjiesu@sjtu.edu.cn (Y.S.); 2 Zhoushan Field Scientific Observation and Research Station for Marine Geo-Hazards, China Geological Survey, Qingdao 266237, China; 3 Department of Ocean Science and Engineering, Southern University of Science and Technology, Shenzhen 518055, China

**Keywords:** gas sensor, covalent organic framework, bimetallic, chemiresistive, phthalocyanine

## Abstract

Two-dimensional (2D) phthalocyanine-based covalent organic frameworks (COFs) provide an ideal platform for efficient and rapid gas sensing—this can be attributed to their regular structure, moderate conductivity, and a large number of scalable metal active centers. However, there remains a need to explore structural modification strategies for optimizing the sluggish desorption process caused by the extensive porosity and strong adsorption effect of metal sites. Herein, we reported a 2D bimetallic phthalocyanine-based COF (COF-CuNiPc) as chemiresistive gas sensors that exhibited a high gas-sensing performance to nitrogen dioxide (NO_2_). Bimetallic COF-CuNiPc with an asymmetric synergistic effect achieves a fast adsorption/desorption process to NO_2_. It is demonstrated that the COF-CuNiPc can detect 50 ppb NO_2_ with a recovery time of 7 s assisted by ultraviolet illumination. Compared with single-metal phthalocyanine-based COFs (COF-CuPc and COF-NiPc), the bimetallic structure of COF-CuNiPc can provide a proper band gap to interact with NO_2_ gas molecules. The CuNiPc heterometallic active site expands the overlap of *d*-orbitals, and the optimized electronic arrangement accelerates the adsorption/desorption processes. The concept of a synergistic effect enabled by bimetallic phthalocyanines in this work can provide an innovative direction to design high-performance chemiresistive gas sensors.

## 1. Introduction

In the process of industrial development, waste gases, such as nitrogen dioxide (NO_2_), sulfides, carbides, and volatile organic compounds (VOCs) gases, will unavoidably be produced. Massive industrial waste gas emissions seriously harm the environment and lead to human diseases [1]. Therefore, it is significant to develop high-efficiency, low-cost, and long-life chemiresistive gas-sensing devices to detect toxic and hazardous gases [2]. In particular, electronic devices based on organic framework materials, a type of material that was firstly developed by Yaghi and co-workers [3] with porous, conductive, and building block module designs, have aroused much interest in gas sensing [4]. Recently, a series of applications have been realized based on conductive organic frameworks’ (metal organic frameworks (MOFs) and covalent organic frameworks (COFs)) electronic devices [5]. For instance, the design of planar two-dimensional (2D) MOFs with highly symmetric conjugated ligands regulates the formation of π–d conjugates to promote the delocalization of charge and conductivity. Despite some important breakthroughs of chemiresistive sensors based on MOFs [6], the metal atoms involved as framework linkages as well as active sites tend to collapse during the sensing process. In contrast, COFs are considered as much more stable sensing materials due to their highly stable covalent linkages formed by Schiff-based and reversible coordination chemistry [7]. Compared to other materials, 2D COFs combined with a large surface area, abundant active sites, and designable and stable characteristics are more attractive. However, the synthetic process of chemiresistive COFs for gas sensor fabrication is much more difficult, owing to their robust covalent linkages [8,9,10]. Hence, it is challenging to develop gas-sensing devices based on such porous, conductive, and stable COFs materials.

Currently, the introduction of metal atoms into the ligands to improve the electrical conductivity of 2D COFs is regarded as a main modification method. The porphyrin/phthalocyanine units with metal heteroatoms are introduced into the backbone of 2D COFs, which can expand the level of conjugation, generating an AA stacking to boost the crystallinity and surface area of COFs. Metal active sites in COFs can be uniformly distributed in the 2D plane due to the periodic structures of porphyrin/phthalocyanine [11]. Previous reports also demonstrate that the ligand metal in the center of the porphyrin/phthalocyanine ring can effectively modulate the carrier mobility of COFs [12,13]. The connecting blocks of octa-amino phthalocyanine in COF-DC-8 can improve the conductivity of COFs (2.51 × 10^−3^ S/m) [9]. Because the core of the phthalocyanine unit may alter conductivity during gas adsorption, COF-DC-8 is also valuable as a sensitive layer for chemiresistive gas sensors [14]. It has been further demonstrated that COF-DC-8 could respond to different reducing and oxidizing vapors, such as NO_2_, NH_3_, and H_2_S, with ultra-low detection limits. The structural diversity derived from the phthalocyanine macrocyclic compounds that can chelate with different metals endow good sensitivity for different analytes [15,16]. An organic heterojunction device based on octachlorinated metal phthalocyanines (M(Cl_8_Pc); M: Co, Cu, and Zn) and lutetium bisphthalocyanines (LuPc_2_) form the bilayers for NH_3_ sensing. It is shown that the LuPc_2_/Zn(Cl_8_Pc) heterojunction behaves as a *p*-type device after preparation, then gradually switches to *n*-type after long-term exposure to NH_3_ at high relative humidity. The polarity transition of the device is attributed to the change in the electron affinity of Zn(Cl_8_Pc) [17]. Hence, the role of the central metal atom in the phthalocyanine is remarkable to adjust the electronic properties of the device. In addition, benefiting from the photosensitive performance of phthalocyanines, the recovery process can be accelerated by ultraviolet and visible spectrum (UV) illumination [18]. The laser is simple to be operated in practical applications with low power levels. A carboxyl-modified cobalt phthalocyanine, for example, has previously been reported for NO_2_ detection. It is explained in the work that a laser-assisted approach can generate electron/hole pairs to promote desorption and improve the conversion from NO_2_^−^ to NO_2_ [19].

Recently, engineering dual-metal atom centers of the COFs with optimized electronic structures is demonstrated to be an effective strategy for boosting sensing and catalytic performance [20]. The design of two metal-linked central building blocks allowed for the generation of dual-metal ion active centers in phthalocyanine-based COFs [21]. Specifically, the interaction between the two metals may optimize gas molecule adsorption and proton–coupled electron transfer, endowing phthalocyanine-based COFs with excellent sensing properties [22]. Therefore, the modulation of the coordination metal central species is an effective way to improve the sensing performance of 2D phthalocyanine-based COFs [23]. In comparison with single metallic sites, the bimetallic sites have more complex local coordination environments, which are attributed to the replacement of metal ion-induced electronic structure changes in COFs [24]. Furthermore, by distributing the bimetallic components equally at the molecular level, it is possible to provide sufficient active sites to significantly enhance the gas-sensing performance. Although great advancements have been achieved for COF-based sensing devices, the coordination metals as well as high surface areas often lead to strong adsorption forces, resulting in serious desorption problems. A key issue has emerged to balance the relationship between adsorption and desorption energies to improve the gas-sensing performance by exploiting the bimetallic effect [25]. It is still challenging to design COF materials with unique structures for fast gas response/recovery.

Herein, 2D chemiresistive COFs based on bimetallic phthalocyanine are demonstrated for efficient NO_2_ detection at the ppb level. Such a 2D bimetallic network provides more favorable reaction centers with enhanced gas-sensing performance. The COF-CuNiPc-based sensor can detect 50 ppb NO_2_ with a recovery time of 7 s assisted by ultraviolet illumination. Ultra-sensitivity is derived from the optimized selection of bimetallic active sites. The bimetallic network structure of COF-CuNiPc can provide a suitable band gap, while the heterometallic active sites expand the overlap of *d*-orbitals and the optimized electronic arrangement accelerates the adsorption/desorption process of gas molecules. This dual effect of bimetallic phthalocyanine can improve the gas-sensing performance, providing a new insight to design effective COF-based gas sensors.

## 2. Materials and Methods

### 2.1. Materials

All chemicals and reagents were commercially available and utilized without additional purification. Dehydrated *N*,*N*-dimethylformide (DMF), triethylamine (Et_3_N), acetone, formaldehyde, CHCl_3_, ethanol, ethyl acetate (EtOAc), n-hexanal, and tetrahydrofuran (THF) were purchased from Admas. Nickel(II) 2,3,9,10,16,17,23,24-octafluorophthalocyanine (NiPcF_8_, building blocks-2) and copper(II) 2,3,9,10,16,17,23,24-octafluorophthalocyanine (CuPcF_8_, building blocks-3) were purchased from Sigma-Aldrich (Shanghai, China). Copper(II) 2,3,9,10,16,17,23,24-octahydroxylphthalocyanine (CuPc[OH]_8_, building blocks-1) and nickel(II) 2,3,9,10,16,17,23,24-octahydroxylphthalocyanine (NiPc[OH]_8_, building blocks-4) were synthesized according to the literature [26,27].

### 2.2. Synthesis of COF-CuNiPc

COF-CuNiPc was prepared by the nucleophilic substitution of the hydroxyl group, according to the previous report [28]. Briefly, a mixture of CuPc[OH]_8_ (20 mmol, 17.28 mg) and NiPcF_8_ (20 mmol, 14.21 mg) was added into a 10 mL Pyrex vial, followed by the addition of 1.0 mL DMF and 0.2 mL Et_3_N as a catalyst. The mixture was sonicated for 5 min to form a homogeneous suspension. The Pyrex vial was degassed via three freeze–pump–thaw cycles and sealed by a flamer, then heated at 100 °C for seven days. The precipitate was collected through centrifugation and completely rinsed with THF until the eluate was colorless. After that, the as-obtained crude product was subjected to Soxhlet extraction with THF as eluate for three days to remove soluble purities. The COF-CuNiPc was collected and dried at 100 °C under a vacuum overnight. To verify the effect of bimetallic phthalocyanines in the topology, CuPc[OH]_8_, CuPcF_8_ (building blocks-1 and blocks-3), and NiPc[OH]_8_, NiPcF_8_ (building blocks-4 and blocks-2) were introduced into COF-CuPc and COF-NiPc according to the same preparation scheme described above.

### 2.3. Characterization

The morphology and crystalline structure of the obtained samples were examined by field-emission scanning electron microscope (FE-SEM, Ultra plus, Carl Zeiss, Oberkochen, Germany), and powder X-ray diffraction (PXRD) measurements (D8 Advance, Bruker, Mannheim, Germany) with Cu Kα source (λ = 0.15418 nm). Fourier transform infrared spectroscopy (FT-IR) spectra were recorded using a Thermo Fisher Nicolet 6700 FT-IR spectrometer (Thermo Fisher, Waltham, MA, USA). The element composition and internal bonding were probed by X-ray photoelectron spectroscopy (XPS, Japan Kratos Axis Ultra DLD, AlKα, 1486.6 eV). UV–vis diffuse reflectance spectroscopy (UV-Vis DRS) spectra were detected by a spectrophotometer (Lamda 950, PerkinElmer, Waltham, MA, USA) with a wavelength range from 200 to 800 nm.

### 2.4. Gas Sensor Fabrication and Sensing Measurements

A drop coating method was applied to decorate COF materials on interdigital electrodes (IDEs) to obtain the chemiresistive COF-based gas sensors [29]. The signal was obtained by an Agilent 4156 C (Santa Clara, CA, USA), maintaining a voltage of 3.0 V during the test. The flow rate of the gas was regulated by mass flow controllers (MFCs) and the carrier gas was compressed air. For selectivity testing of the gas sensor, standard NO_2_ cylinders were replaced by corresponding standard cylinders including H_2_, NH_3_, acetone, formaldehyde, CHCl_3_, ethanol, EtOAc, and n-hexanal. The effect of humidity on the gas sensor was tested at 20% to 80% relative humidity (RH) by a simple bubbling method [30]. The response of the gas sensor at room temperature (RT) was defined as Equation (1):

Response = ∆*I*/*I*_a_ × 100% = (*I*_g_ − *I*_a_)/*I*_a_ × 100% (1)

where *I*_a_ is the current value of the gas sensor after stabilization in air, and *I*_g_ is the resistance value of the gas sensor after exposure to NO_2_ for a certain time. In the experiment, the wavelength of the UV light-emitting diode (LED) light used was 365 nm. The power of the selected UV light was 0.15 W. The effective intensity of UV irradiation at a distance of 3 cm between the gas sensor and the UV light was 7.07 × 10^−4^ W/m^2^.

## 3. Results

### 3.1. Synthesis and Characterization of COFs

**Figure 1:**
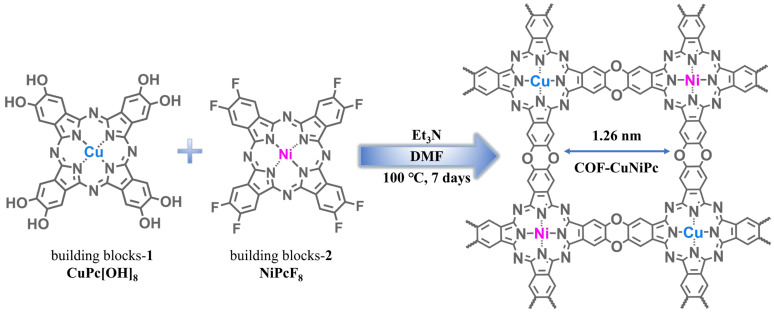
Design and synthesis of COF-CuNiPc.

In this study, we developed three kinds of phthalocyanine-based COFs using CuPc[OH]_8_, NiPcF_8_, CuPcF_8_, and NiPc[OH]_8_ as building blocks via the nucleophilic aromatic substitution reaction (Figure 1). The hydroxyl groups were nucleophilically substituted by perfluorinated electron-deficient phthalocyanine, resulting in quantitative dibenzo-p-dioxin production. The simultaneous incorporation of Cu and Ni into bimetal COF-CuNiPc broke the symmetry of the active site down to the synergistic effect of the adsorption/desorption process [31]. The *C*_4_ + *C*_4_ topological diagram suggests that these COFs should have well-aligned 2D sheets and uniform channels with theoretical pore sizes of 1.26 nm. To verify the effect of bimetallic phthalocyanines in the topology, the corresponding model compound and synthetic routes are shown in Figures S1 and S2.

**Figure 2:**
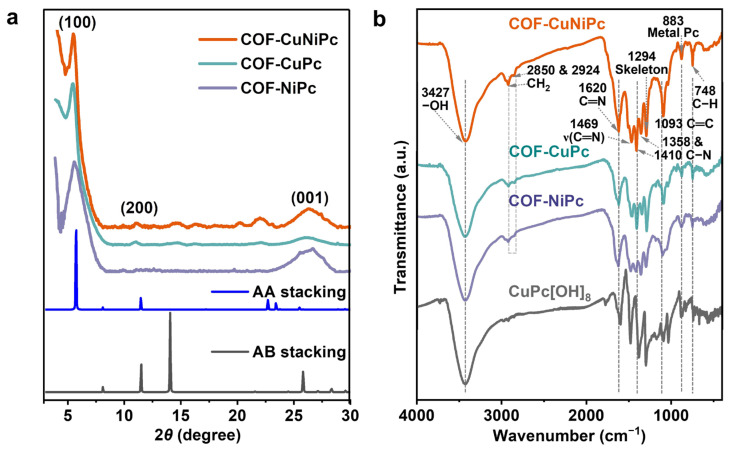
(**a**) PXRD patterns of COF-CuNiPc (orange curve), COF-CuPc (green curve), COF-NiPc (purple curve), AA stacking (blue curve), and AB stacking (grey curve). (**b**) FT-IR spectra of COF-CuNiPc, COF-CuPc, COF-NiPc, and CuPc[OH]_8_.

The chemical characteristics and crystal structures of COF-CuNiPc, COF-CuPc, and COF-NiPc were investigated by PXRD and FT-IR techniques. The PXRD of COF-CuNiPc, COF-CuPc, and COF-NiPc measurements were assisted by the computational simulation calculation. As shown in the orange curve in Figure 2a, COF-CuNiPc exhibits a series of strong PXRD peaks at 5.58°, 11.14°, and 26.21°, which are assigned to (100), (200), and (001) facets, respectively [32]. Both COF-CuPc and COF-NiPc also exhibit extremely similar pattern profiles, demonstrating that the synthesized 2D phthalocyanine COFs have good crystalline structures. The PXRD patterns of AA stacking (Figure 2a, blue line) and AB stacking (Figure 2a, grey line) patterns are assisted by the Forcite geometric simulation method [33]. The results show that the AA stacking virtually replicates the experimental PXRD patterns of phthalocyanine COFs. Consequently, the AA stacking mode promotes orbital overlap in the association of periodic crystallization and porous structures, which improves the surface area and helps to achieve significant sensing performance [34,35]. FT-IR measurements further verify the successful preparation of the 2D phthalocyanine COFs with the characteristic absorption peaks identified in Figure 2b. The result shows the typical –OH absorption peaks around 3427 cm^−1^. The characteristic peaks at 1093 and 1294 cm^−1^ are attributed to the vibrations of the phthalocyanine backbones [36]. Furthermore, the adsorption peak at 1620 cm^−1^ belongs to C=N stretching, which proves the existence of phthalocyanine backbones [37]. The strong peaks found at about 1358 and 1410 cm^−1^ are attributed to ν(C=N–C=C), and the peak at 1469 cm^−1^ is ascribed to ν(C=N) [38]. A weaker peak also appears at 883 cm^−1^, which is contributed to the phthalocyanine metal–N bond, indicating that it is still stable in the topological ring of COFs [39]. The characteristic peak present at 748 cm^−1^ belongs to the benzene ring substitution adjacent to the carbon–hydrogen stretching absorption peak of C–H [40].

**Figure 3:**
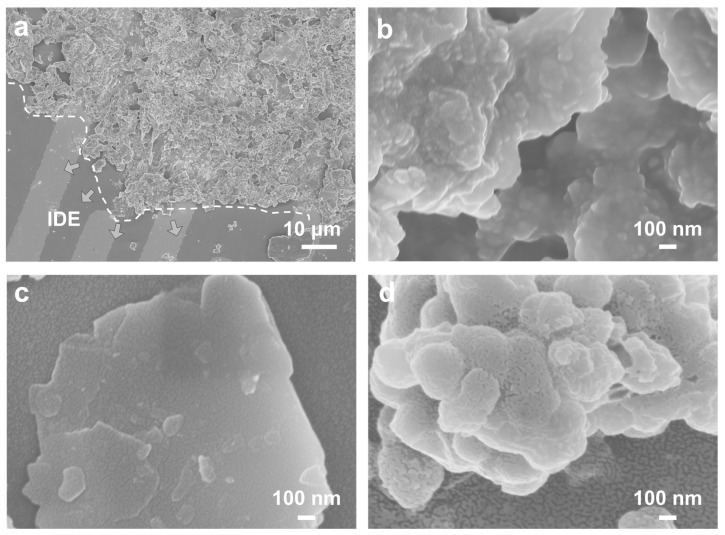
SEM images of COF-CuNiPc with a scale bar of (**a**) 10 μm (the direction of the arrows represent the substrate morphology of the IDE’s surface), (**b**) 100 nm, (**c**) COF-CuPc, and (**d**) COF-NiPc on IDE’s substrate.

The surface morphology of COFs on the IDE substrate was investigated by SEM. Figure 3a,b illustrate the SEM images of COF-CuNiPc deposited on IDE substrate. It can be seen from Figure 3a that the powder looks like a stacked structure of sheet layers. As for the monomer CuPc[OH]_8_, its morphological analysis was also performed (Figure S3). Unlike COF-CuNiPc, the drop-coated CuPc[OH]_8_ on the substrate of IDEs shows larger particles. Similar porous sheets stacked morphology in the SEM images of COF-CuPc, and COF-NiPc deposited on IDEs can be also observed. The sensing layers of the devices based on COF-CuNiPc, COF-CuPc, and COF-NiPc are 61.3, 43.8, and 55 μm, respectively (Figure S4). The porosities of the COF-CuNiPc and CuPc[OH]_8_ were investigated by nitrogen sorption measurements at 77 K. As shown in Figure S5, the Brunauer–Emmett–Teller (BET) surface area of COF-CuNiPc was found to be 624.36 m^2^ g^−1^, which shows a higher value compared to that of CuPc[OH]_8_ (90.99 m^2^ g^−1^). It is assumed that this larger surface area can lead to a better sensing adsorption space for NO_2_, which can be also confirmed in the porous morphology by SEM characterizations.

### 3.2. Gas-Sensing Properties of the COF-Based Devices

**Figure 4:**
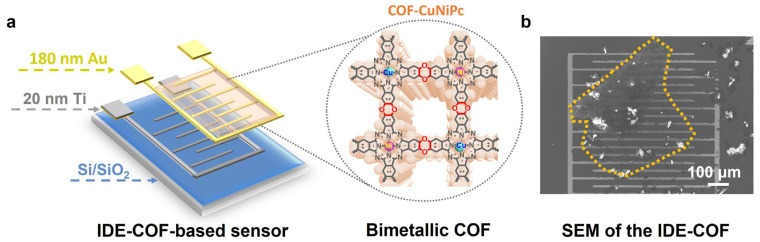
The (**a**) structure of the IDE sensor based on COF-CuNiPc (the gold, gray and blue dashed lines indicate the type and thickness of the IDE sputtering layer materials; a magnified view of the COF-CuNiPc structure on the IDE is shown in the gray circle). (**b**) SEM images of the IDE sensor based on COF-CuNiPc (the golden dashed area represents the COF material spread on the surface of IDE).

IDEs are obtained by magnetron sputtering of a 180 nm gold layer and a 20 nm titanium layer on a Si/SiO_2_ substrate (Figure 4a). A drop-coating approach is used to combine COF-CuNiPc with IDEs in the chemiresistive gas sensor [41]. The COF-CuNiPc material was uniformly dispersed in the ethanol solution via sonication treatment. The as-formed COF suspension was drop-coated on the hydrophilic-treated IDE surface and dried to form a conductive circuit. The SEM image (Figure 4b) displays that COF-CuNiPc materials are deposited on the substrate and bridged between the IDE. The gas-sensing tests are performed in the system presented in Figure S6. There is a gas distribution chamber that includes a gas inlet, test chamber, and gas outlet. The gas inlet and outlet were set as a through-hole with a diameter of 4 mm. A cylindrical body with an inner diameter (*d*) of 2 cm, a height (*h*) of 1.7 cm, and a volume (*V* = π(*d*/2)^2^ × *h*) of 5.34 cm^3^ was used as the test chamber.

Before gas testing, *I*–*V* curves of the devices were first tested under bias voltage to ensure device connectivity. Then, the dry, compressed air was introduced into the test chamber for 2 min to guarantee a reliable signal output from the devices. The results are shown in Figure S7, and gas-sensing devices based on both the building blocks and 2D phthalocyanine COFs can exhibit better conductivity compared to other organics. The sensing properties of phthalocyanine building blocks were investigated to compare with the synthesized COFs.

**Figure 5:**
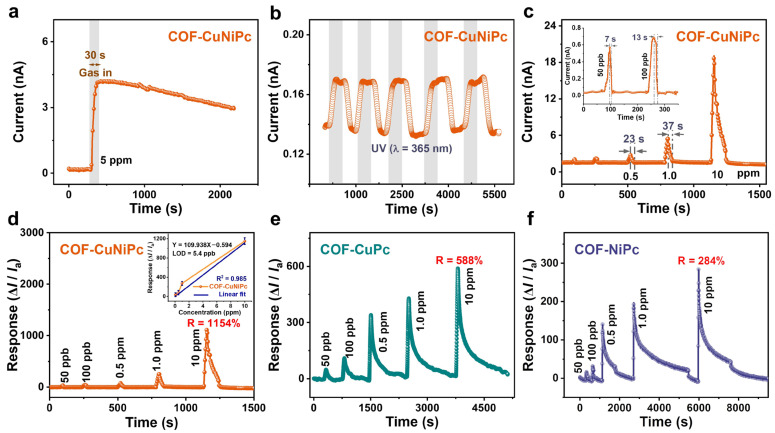
(**a**) Dynamic current characteristic curve of the COF-CuNiPc-based sensor exposed to 5 ppm NO_2_ (without light irradiation). (**b**) Response of COF-CuNiPc toward UV light (365 nm) without NO_2_ at RT. (**c**) Dynamic current characteristic curve of the COF-CuNiPc-based sensor exposed to different concentrations of NO_2_ (0.05, 0.1, 0.5, 1.0, and 10 ppm) with UV-assisted recovery. Dynamic response characteristic curves of (**d**) COF-CuNiPc (the inset shows the linear fit curve of response versus the concentration), (**e**) COF-CuPc, and (**f**) COF-NiPc-based sensors exposed to different concentrations of NO_2_ with UV-assisted recovery.

The dynamic response curves of sensing devices based on COF-CuNiPc with building blocks of CuPc[OH]_8_ and NiPcF_8_ exposed to 1 ppm NO_2_ are shown in Figure S8. We can see that the gas sensor with CuPc[OH]_8_ exhibited better electrical conductivity than the device containing NiPcF_8_, which is consistent with the *I*–*V* curve test results. In addition, the gas sensor with CuPc[OH]_8_ could respond to NO_2_, while the NiPcF_8_-based device did not show any response signal. We speculate that the coordination metal species and hydroxyl functional groups of CuPc[OH]_8_ play a key role in the COF-CuNiPc-sensing performance [42]. However, the CuPc[OH]_8_-based sensor could not recover after the first three detection cycles. Indeed, the current signal was still ascending when the gas purging was switched off for the fourth time to detect NO_2_, suggesting that poisoning of the sensitive material had occurred. A continuous response (>100 min) was performed for CuPc[OH]_8_ and COF-CuNiPc fluxed with 100 ppb NO_2_, and both exhibited response signals that could not be saturated (Figure S9). Therefore, as for experimental normalization in the later sensing experiments, the exposure time of the control gas will be fixed at 30 s in order to compare the sensing performance of COF materials. Since phthalocyanine acts as a good photosensitizer [43], the recovery performance of the sensors based on phthalocyanine monomer and COFs was boosted by the UV-illumination method. The absorption spectrum of COF-CuNiPc was tested by solid UV–vis DRS before using light-assisted recovery. Based on the UV–vis DRS results, the appropriate wavelength of light was selected to optimize performance. The spectra showed that COF-CuNiPc had distinct absorption peaks at 345 and 384 nm, so 365 nm was chosen as the light source of assisted desorption (Figure S10). We subsequently used a UV-assisted method to improve the recovery performance of CuPc[OH]_8_-based sensors (Figure S11). Illumination of the light only increased the carrier concentration of CuPc[OH]_8_ to improve conductivity, which did not produce a full desorption effect of the gas. Furthermore, to exclude the potential effect of illumination on the current signal of the COF-CuNiPc-based sensor, the UV exposure cyclic response was tested under the conditions of compressed air without NO_2_ as a background. As shown in Figure 5b, the COF-CuNiPc-based device exhibits a stable, periodic current-positive dynamic response curve under UV illumination. Regarding the recovery of the COF-CuNiPc-based sensor exposed to NO_2_, the current signal exhibits a gradually decreasing tendency. Thus, there is no effect on the intrinsic gas-sensing performance of the COF-based sensors to the NO_2_ gas without light irradiation.

**Figure 6:**
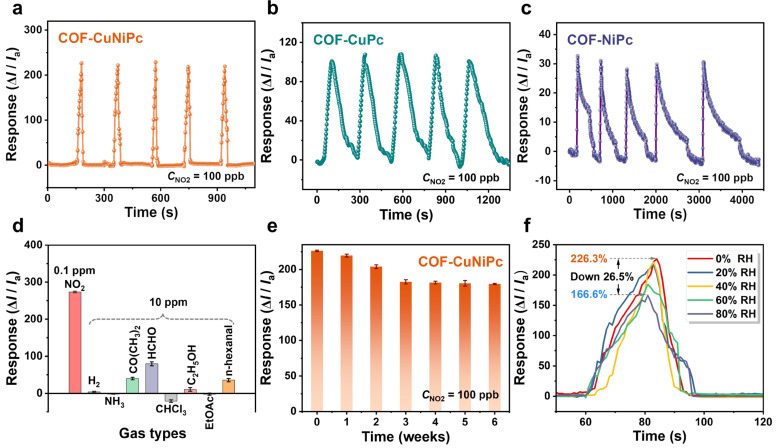
The response and recovery curves for five cycles of the gas sensors based on (**a**) COF-CuNiPc, (**b**) COF-CuPc, and (**c**) COF-NiPc exposed to 100 ppb NO_2_. (**d**) Real-time current curves of COF-CuNiPc-based gas sensors against different vapors. (**e**) The long-term response stability of the COF-CuNiPc-based sensor. (**f**) The dynamic response characteristic curves of COF-CuNiPc-based sensors exposed to 100 ppb NO_2_ at 20, 40, 60, and 80% RH.

As shown in Figure 5, the results of the sensing behavior of sensors based on bimetallic COF-CuNiPc, monometallic COF-CuPc, COF-NiPc, as well as the monomer CuPc[OH]_8_ for NO_2_ gas were investigated. All devices exhibit the sensing characteristics of *p*-type semiconductor chemoresistance, with the resistance of the sensor decreasing when exposed to the oxidizing gas NO_2_, and conversely recovering in the air [44]. Distinctly, the bimetallic COF-CuNiPc exhibited outstanding sensing performance, with experimental tests requiring only 7 s for complete gas desorption with UV light assistance after 30 s of exposure to 50 ppb NO_2_. The recovery times of the COF-CuNiPc-based sensor exposed to 0.1, 0.5, and 1.0 ppm NO_2_ were 13, 23, and 37 s, respectively. In addition, the response value of the sensor based on COF-CuNiPc exposed to 10 ppm NO_2_ could even reach up to 1154%. A linear fit of the response value ratio to NO_2_ concentration showed that a theoretical limit of detection (LOD) of NO_2_ was 5.4 ppb for the gas sensor based on COF-CuNiPc [45]. The LOD value of the devices based on monometallic COF-CuPc and COF-NiPc were, respectively, 191.5 and 244.8 ppb for NO_2_ (Figure S12). The fitted equations for monometallic COFs both had low squared differences and showed nonlinear relationships. In addition, the building blocks of CuPc[OH]_8_ could not be fully recovered, although UV-illumination-assisted gas desorption was carried out. As for monometallic COF-CuPc and COF-NiPc, the response values of 588% and 284% at 10 ppm NO_2_ are inferior to the bimetallic COF-CuNiPc (Figure 6e,f), although these comparative monometallic phthalocyanine-based sensors can fully recover. In addition, both recovery times of COF-CuPc and COF-NiPc are much longer than that of the bimetallic COF-CuNiPc, highlighting the key role of bimetallic designing in gas-sensing enhancement.

To investigate the repeatability of the phthalocyanine 2D COF-based devices, the sensing devices were kept in a chamber and cycled with the introduction of 100 ppb NO_2_ gases. The results show that gas sensors based on both COF-CuNiPc and COF-CuPc exhibit stable recyclability, while the response of COF-NiPc-based sensing devices decreases slowly with a gradually increasing recovery time (Figure 6a–c). Comprehensively, the bimetallic COFs-CuNiPc showed remarkable performance in sensitivity, fast recovery, and cycling stability. Accordingly, we further investigated the interference resistance of COF-CuNiPc devices. The results show that COFs-CuNiPc has good selectivity to NO_2_ and maintains stable sensing performance over a period of up to six weeks. Generally, the humidity in the air can affect the sensitivity, so the gas-sensing performance of the devices exposed to 100 ppb NO_2_ under different humidities was studied. At 80% RH, the response value of the device decreases by 26.5% compared to the response value at a completely dry 0% RH, with a response value of 166.6%. The performance of the bimetallic COF-CuNiPc-based sensor was compared with other reported works; the results are shown in Table 1. We can see that a superior performance has been achieved for our COF-CuNiPc-based gas sensors, highlighting their great potential for gas sensing.

## 4. Discussion

**Figure 7:**
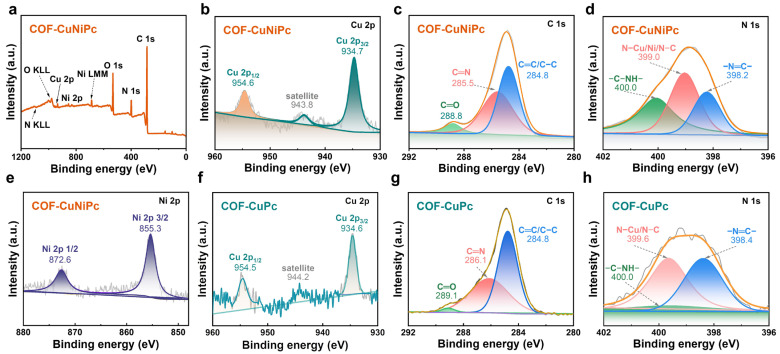
(**a**) XPS survey spectrum for COF-CuNiPc. High-resolution (**b**) Cu 2p, (**c**) C 1s, (**d**) N 1s, and (**e**) Ni 2p core-level XPS spectra of COF-CuNiPc. High-resolution (**f**) Cu 2p, (**g**) C 1s, and (**h**) N 1s core-level XPS spectra of COF-CuPc.

To probe the gas-sensing mechanism of 2D phthalocyanine-based COFs, XPS and UV–vis DRS were measured to investigate the elemental composition, metal valence, and band gap of the 2D phthalocyanine COFs. All elements of COF-CuNiPc, including Cu, Ni, C, N, and O, can be observed in the spectra (Figure 7a). The high-resolution C 1s, N 1s, and O 1s XPS spectra of COF-CuNiPc and COF-CuPc show that their binding energy peaks are similar (Figure 7). Among them, the O 1s signal of COF-CuNiPc displays two peaks at 532.3 and 533.4 eV, which can be attributed to the C–O–C bond of the aromatic group and the atmospheric adsorption of H_2_O/CO_2_, respectively, while only a single peak was observed in CuPc[OH]_8_ (Figure S13) [55]. The high-resolution Cu 2p belonging to the COF-CuNiPc spectrum contains two distinct signal peaks at 934.7 and 943.8 eV, assigned as Cu 2p_1/2_ and Cu 2p_3/2_, where there is also a weaker instrument-generated satellite peak at 954.6 eV [56].

The core-level spectrum of Ni 2p belonging to COF-CuNiPc XPS spectrum is located at 855.3 eV (Ni 2p_3/2_) and 872.6 eV (Ni 2p_1/2_), corresponding to Ni(II) [57]. The XPS spectra of C 1s attributed to COF-CuNiPc can be divided into three characteristic binding energy peaks located at 284.8, 285.5, and 288.8 eV, corresponding to C=C/C–C, C=N, and C=O, respectively [58]. After the condensation reaction with phthalocyanine monomers, those N 1s binding energy peaks of COF-CuNiPc (–N=C– and –C–NH–) shift to higher binding energy values at 398.4, 399.6, and 400.0 eV [59]. Interestingly, N 1s would be shifted by the binding energy peak position of COF-CuNiPc at 399.0 eV (N–Cu/Ni/N–C) because of the different coordination metals in the phthalocyanine framework, and the binding energy peak of N–Cu/N–C of COF-CuPc is 399.6 eV. Comparing bimetallic COF-CuNiPc (Figure 7b) and monometallic COF-CuPc (Figure 7f), the main Cu 2p_3/2_ and Cu 2p_1/2_ peaks in bimetallic COF-CuNiPc exhibited higher binding energy (positive shift of about 0.1 and 0.1 eV, respectively), indicating that the synergistic effect of bimetallic COF-CuNiPc drives the electron transfer from Cu to Ni [60].

Furthermore, the possible interactions between the phthalocyanine-based COFs and the monomer with the gas molecules were analyzed by the results of the absorption spectra presented by UV–vis DRS. These materials all have significant absorption peaks in the absorption wavelength range of 300–400 nm, suggesting that UV illumination with the wavelength in this range can offer the most efficient desorption and recovery energy. This is consistent with the test and characterization results mentioned supra. The absorption edges of the three COFs are red-shifted due to the presence of the conjugate structure (π→π* transition) of the COFs. In this case, the spectral results of COF-CuNiPc and COF-CuPc would be more prominent, suggesting that the core metal differences of the framework lead to the altered absorbance and emission of the COF-based materials [61]. The corresponding band gaps of phthalocyanine-based COFs are determined by the known UV–vis-based Tauc-plot method with Equation (2):

(2) αhν=hν−Eg1n,

where *α* is the absorption coefficient, *hν* is the photon energy, *h* is Planck’s constant, *ν* is the frequency of light, and *n* denotes the indirect (*n* = 2) or direct (*n* = 1/2) band gap material [62]. The intersection of the tangent line at the maximum slope of the Tauc plot with the transverse coordinates was calculated for the band gap of 1.814, 1.674, 2.26, and 2.328 eV for COF-CuNiPc, COF-CuPc, COF-NiPc, and CuPc[OH]_8_, respectively. Due to their regularly oriented ion transfer channels and continuous conduction sites, phthalocyanine-based COFs exhibit a smaller band gap than monomers to achieve a superior performance in chemiresistive gas sensing.

We assume that the chemiresistive-sensing mechanism of the NO_2_ by bimetallic COF-CuNiPc arises from the gas analyte being adsorbed on the metal active center to induce interactions. As the COF material is exposed to the target gas atmosphere, such a continuous charge transfer or redox would potentially trigger a change in the conductivity of the sensitive material [27]. Thus, we capture the change in the electrical signal output from the COF-based sensor for gas sensing. When exposed to a NO_2_ atmosphere with a strong electron acceptor, it binds to the metal atom center of the COFs in the forms of NO_2_^−^ and NO^−^. Subsequently, COF is accompanied by UV irradiation to produce electron–hole pairs again, prompting it to complete the NO_2_ desorption process. According to the conventional phthalocyanine-sensing process [63], the desorption reaction will occur according to Equation (3):

(3) $$2NO_{3}^{-}\overset{h\nu_{<420nm}}{\to }2NO^{-}+O_{2}^{-}+e^{-},$$

Therefore, the phthalocyanine-sensitive layer is often oxidized gradually by atomic oxygen in the process, leading to the toxicity of the sensor. In contrast, the COF-based structure can tackle this issue, owing to its stable topology formed by fully covalent reversible coordination chemistry. This avoids the oxidation of the sensing material and reduces the thermal deactivation of the photoexcited state caused by rotation. Phthalocyanine-based COFs with ordered pores and regular coordination metal active centers become suitable candidates for electron-deficient NO_2_ sensing [64].

**Figure 8:**
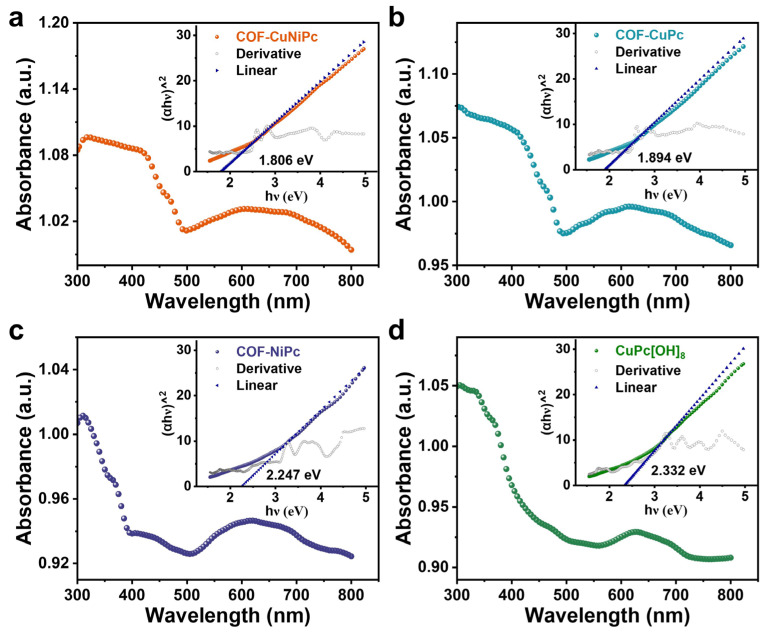
Solid-state absorption spectra of (**a**) COF-CuNiPc, (**b**) COF-CuPc, (**c**) COF-NiPc, and (**d**) CuPc[OH]_8_ as powders using a diffuse reflectance accessory.

**Figure 9:**
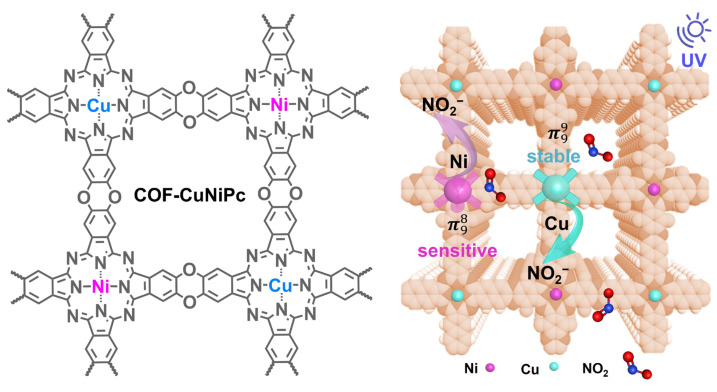
A schematic illustration of the adsorption and desorption of NO_2_ on bimetallic COF-CuNiPc.

The comparison of the band gaps of 2D phthalocyanine-based COFs (Figure 8e) further confirms that even small changes in the coordination metal at the atomic level can lead to large macroscopic differences, leading ultimately to a different sensing performance [65]. The mechanism of the adsorption and desorption of NO_2_ on bimetallic COF-CuNiPc is shown in Figure 9. The proper coordination metal can provide a suitable band gap for 2D phthalocyanine-based COFs to achieve high sensitivity and a fast recovery process. The bimetallic COFs can effectively enhance the sensing performance through the synergistic effect of metal active centers [66]. In the sensing process of this work, intimate contact with NO_2_ molecules is inhibited by limiting the accessibility of protons and electrons to the active sites to speed up the sensor recovery. The central ion Ni^2+^ of [Ni(CN)_4_]^2−^ also has a remaining empty 4*p*_z_ orbital in the vertical plane, which is more accessible to NO_2_ interactions. This is the reason that the gradual increase in the cycle recovery process time occurs for sensors based on COF-NiPc. As we know, [Cu(CN)_4_]^2−^ contains a *d*^9^ structure. The 4*p* orbital of the central ion Cu^2+^, which does not participate in the hybridization, forms $\pi_{9}^{9}$ off-domain large bonds with the π orbitals of four CN^−^, resulting in improvement in the stability [67]. Cu-Ni bimetallic coordination enlarges the overlap of *d*-orbitals, and the resulting heterometallic active sites help to optimize the electronic structure of COFs [68]. Hence, the simultaneous introduction of Cu and Ni into COFs breaks the metal active site electron density symmetry [69] and achieves a fast adsorption-/desorption-sensing process.

## 5. Conclusions

In summary, bimetallic phthalocyanine-based COFs have been reported for high-performance NO_2_ chemiresistive gas sensing. Based on the heterometallic active sites of bimetallic phthalocyanine (e.g., COF-CuNiPc), the band gap of COFs is optimized, and the electronic arrangement can be rationally assigned to create a rational environment for enhancing the response as well as boosting the gas adsorption/desorption of NO_2_ gas molecules. The testing results reveal that the recovery time of the as-designed COF-CuNiPc-based sensor exposed to 50 ppb NO_2_ is 7 s with the assistance of UV illumination, and the LOD can reach 5.4 ppb. XPS and UV–vis DRS partially confirm that the ligand bi-metal species in phthalocyanine-based COFs significantly influenced their performance. This synergistic effect of bimetallic active centers can provide an innovative route for the development of high-performance chemiresistive gas-sensing devices.

## Figures and Tables

**Table 1:** Comparison of the performance of various NO_2_ sensors under UV illumination reported in the literature and the as-fabricated sensor in this work.

Sensing Materials	*C* (ppm)	T (°C)	Response/Recovery Time (s)	Response (%)	LOD (ppb)	Ref.
MoS_2_	100	RT	29/350 (Y)	35.16 (Y) ^1^	—	[46]
MoTe_2_	1	RT	300/120 (Y)	18 (Y) ^2^	0.252	[47]
Graphene	100	RT	200/1000 (Y)	26 (Y) ^1^	42.18	[48]
MoS_2_/GaN	50	RT	184/369 (Y)	98.3 (Y) ^1^	—	[49]
ZnO/MoS_2_	10	RT	~258/~72 (Y)	293 (Y) ^1^	200	[50]
InSe	1	RT	233/350 (Y)	190 (Y) ^1^	0.98	[51]
Ti_3_C_2_T_x_-ZnO	20	RT	22/10 (Y)	78.6 (N) ^1^	50	[52]
MOF (NH_2_-UiO-66)	10	150	~50/~50 (N)	7.6 ± 0.4 (N) ^1^	—	[53]
MOF-Pt/In_2_O_3_	1	40	~900/420 (N)	44.9 (N) ^1^	0.1	[54]
COF-CuNiPc	0.1	RT	30/13 (Y)	226.3 (N) ^3^	5.4	This work

With UV or not: Yes (Y); No (N); ^1^ Response% = (*R*_g_ − *R*_0_)/*R*_0_ × 100%; ^2^ Response% = (*G* − *G*_0_)/*G*_0_ × 1000%; ^3^ Response% = (*I*_g_ − *I*_a_)/*I*_a_ × 100%.

## Data Availability

Not applicable.

## Supplementary Materials

The following supporting information can be downloaded at: https://www.mdpi.com/article/10.3390/nano13101660/s1. Figure S1: Synthetic route and representative structure of COF-CuPc; Figure S2: Synthetic route and representative structure of COF-NiPc; Figure S3: SEM images of CuPc[OH]_8_ with a scale bar of (a) 100 μm and (b) 200 nm on the IDE substrate; Figure S4: Optical images of the cross-sectional view of the IDE sensors based on (a) COF-CuNiPc, (b) COF-CuPc, and (c) COF-NiPc; Figure S5: Nitrogen sorption isotherm curves and the pore size distribution of (a) COF-CuNiPc and (b) CuPc[OH]_8_; Figure S6: Schematic diagram of the NO_2_ sensor test system; Figure S7: *I*–*V* curves of the (a) COF-CuNiPc, (b) COF-CuPc, (c) COF-NiPc, (d) CuPc[OH]_8_, and (e) NiPcF_8_-based chemiresistive device under ±3 V bias in a dry compressed air atmosphere at RT; Figure S8: (a) The 1st, 2nd, 3rd, (b) 4th, and (c) UV-light-assisted dynamic response characteristic curves of CuPc[OH]_8_ (building block-1)-based chemiresistive device exposed to 1 ppm NO_2_ for 30 s at RT. (d) Dynamic response characteristic curves of NiPcF_8_ (building block-2)-based device; Figure S9: (a) Dynamic response characteristic curves of CuPc[OH]_8_-based device, and (b) COF-CuNiPc-based chemiresistive device exposed to 100 ppb NO_2_ for a long time (>6000 s) at RT; Figure S10: The absorption peak positions of COF-CuNiPc were differentiated once with the assistance of the absorption spectrum of UV–vis DRS; Figure S11: Dynamic current characteristic curve of CuPc[OH]_8_-based sensor exposed to different concentrations of NO_2_ (0.05, 0.1, 0.5, 1.0, and 10 ppm) with UV-assisted recovery; Figure S12: The linear fit curve of response versus the concentration absorption for the gas sensors based on (a) COF-CuPc and (b) COF-NiPc; Figure S13: High-resolution O 1s core-level XPS spectra of (a) COF-CuNiPc and (b) CuPc[OH]_8_.
