# Supplementary material for: Two-Dimensional Bimetallic Phthalocyanine Covalent-Organic-Framework-Based Chemiresistive Gas Sensor for ppb-Level NO2 Detection
Source: Nanomaterials (Basel). 2023 May 17;13(10):1660. doi: 10.3390/nano13101660 (PMC10224514; doi:10.3390/nano13101660)
Supplement: Supplementary file 1 [file nanomaterials-13-01660-s001.zip › nanomaterials-2380580-supplementary.pdf]

Supporting Information

Two-dimensional bimetallic phthalocyanine covalent organic framework-based chemiresistive gas sensor for ppb-level NO_2 detection

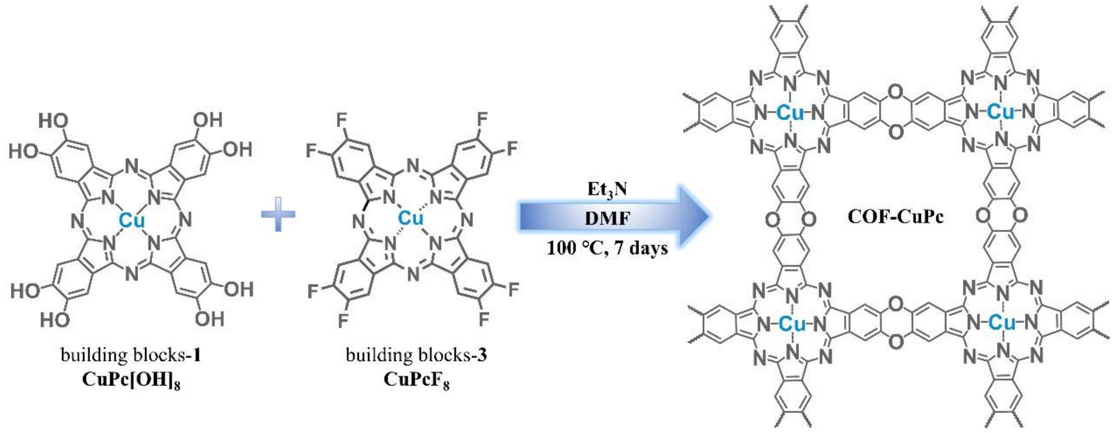

Figure S1. Synthetic route and representative structure of COF-CuPc.

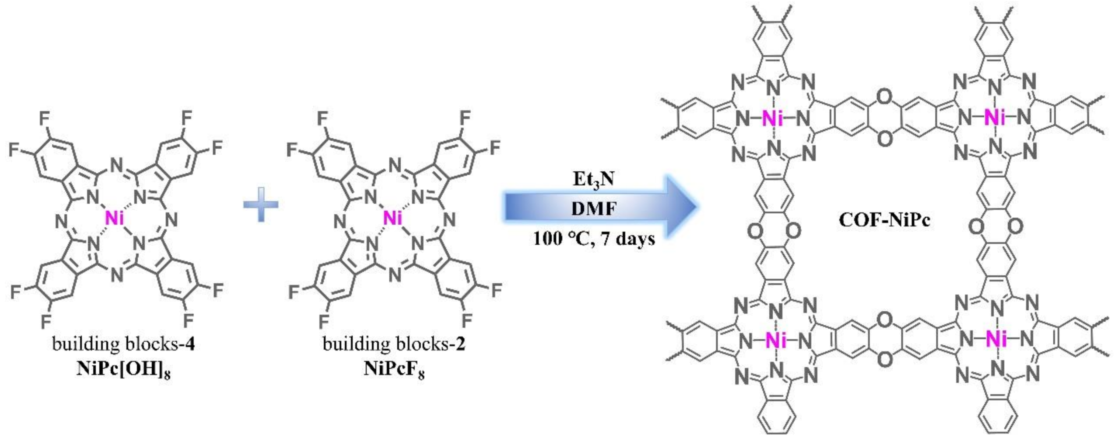

Figure S2. Synthetic route and representative structure of COF-NiPc.

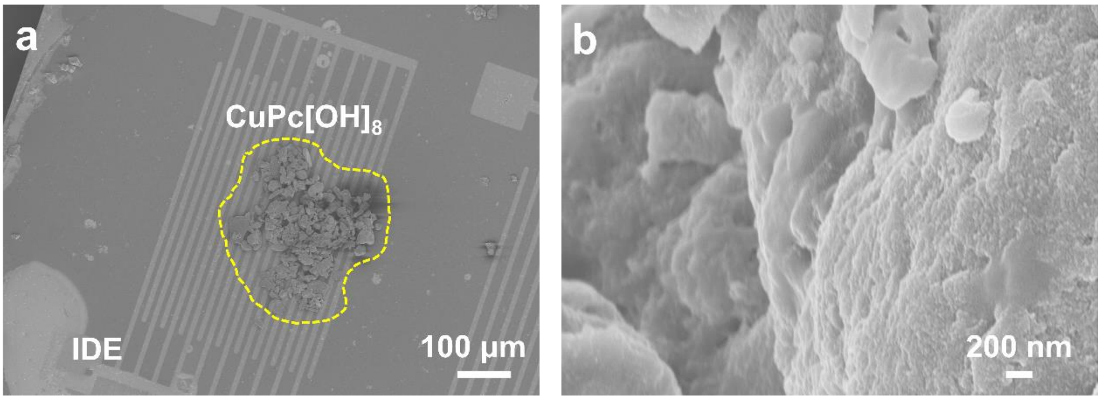

Figure S3. SEM images of CuPc[OH]8 with a scale bar of (**a**) 100 μm and (**b**) 200 nm on IDEs substrate.

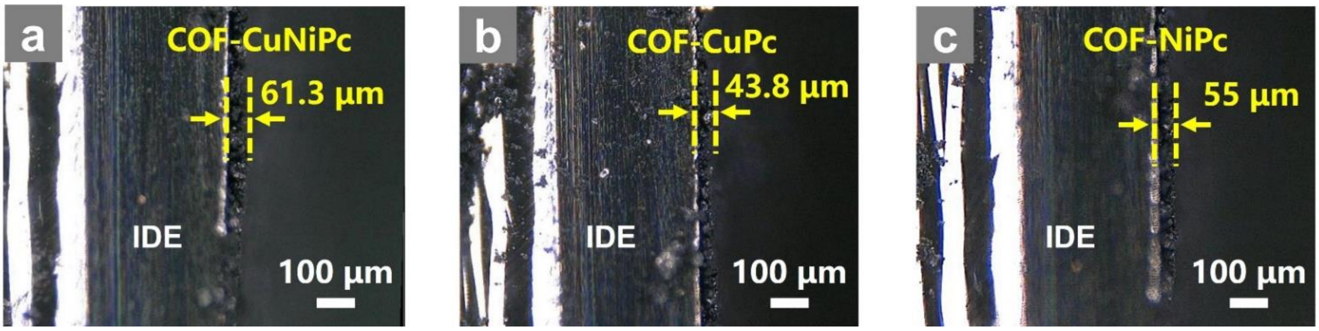

Figure S4. Optical images of the cross-sectional view of the IDE-sensors based on (**a**) COF-CuNiPc, (**b**) COF-CuPc, and (**c**) COF-NiPc.

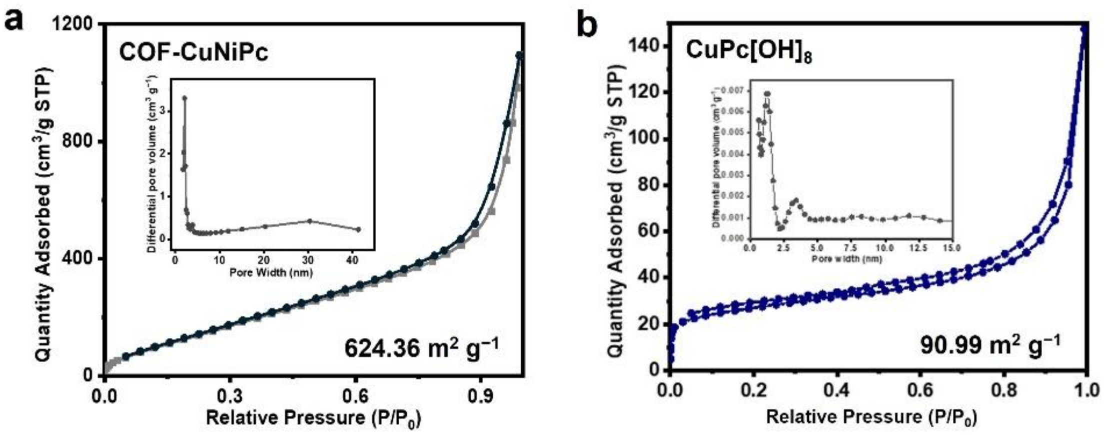

Figure S5. BET Nitrogen sorption isotherm curves and pore size distribution of (**a**) COF-CuNiPc and (**b**) CuPc[OH]8.

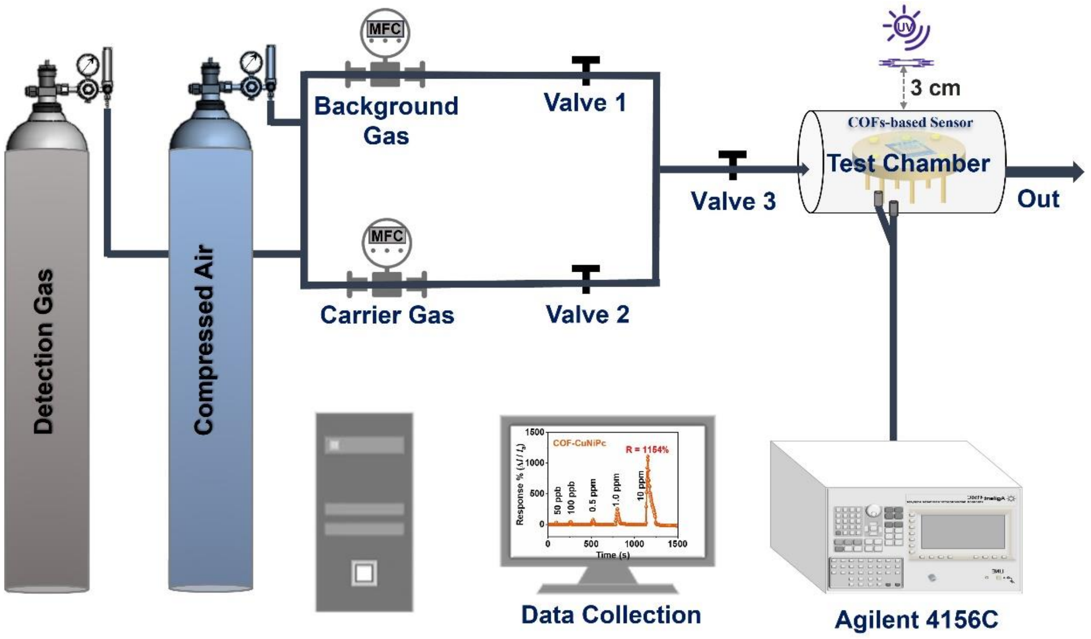

Figure S6. Schematic diagram of the NO_2 sensor test system.

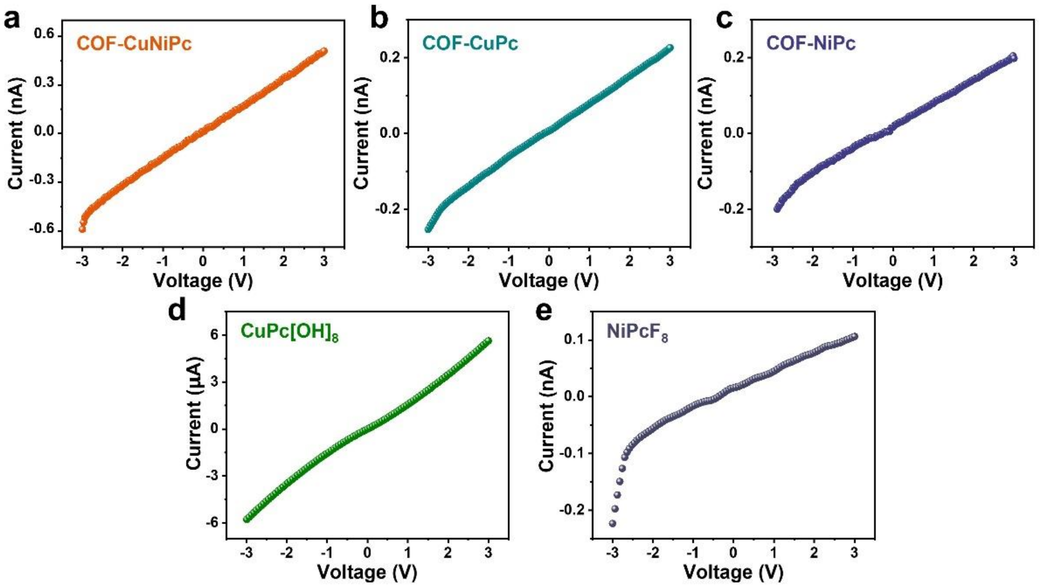

Figure S7. *I-V* curves of the (**a**) COF-CuNiPc, (**b**) COF-CuPc, (**c**) COF-NiPc, (**d**) $CuPc[OH]_8$, and (**e**) $NiPcF_8$-based chemiresistive device under ± 3 V bias in a dry compressed air atmosphere at RT.

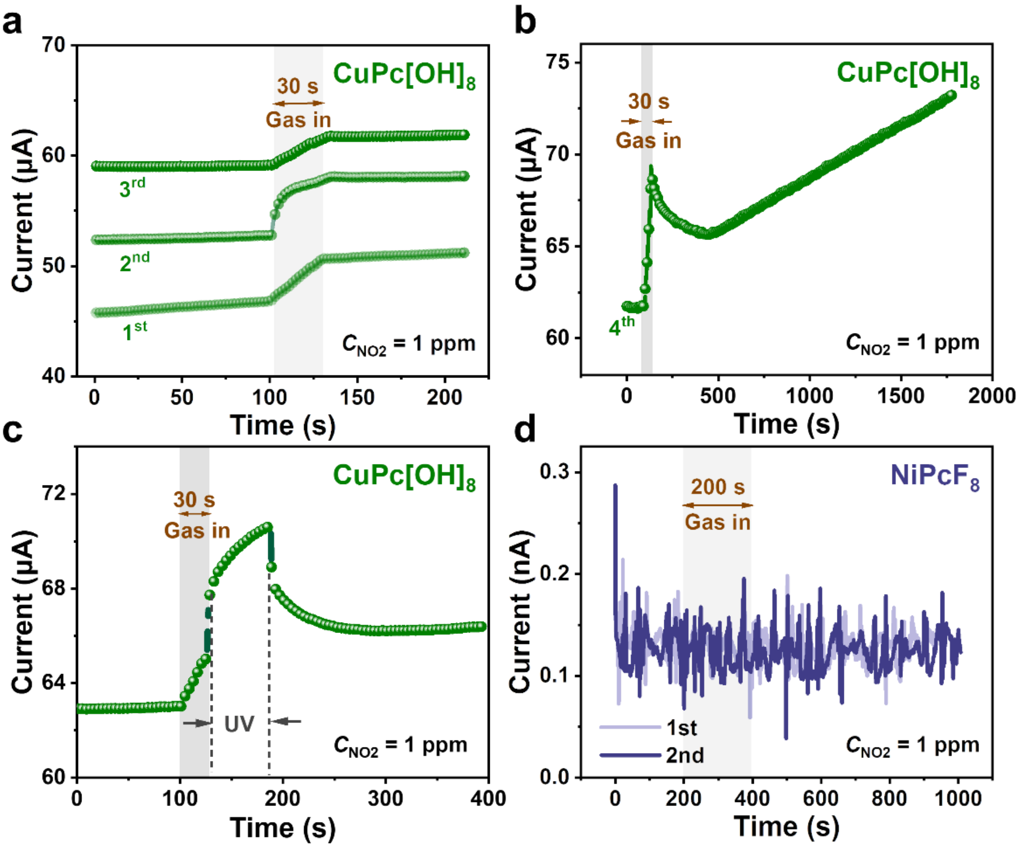

Figure S8. (**a**) The 1st, 2nd, and 3rd, (**b**) 4th, and (**c**) UV-light-assisted dynamic response characteristic curves of CuPc[OH]8 (building block-**1**)-based chemiresistive device exposed to 1 ppm NO_2 for 30 s at RT. (**d**) Dynamic response characteristic curves of $NiPcF_8$ (building block-**2**)-based device.

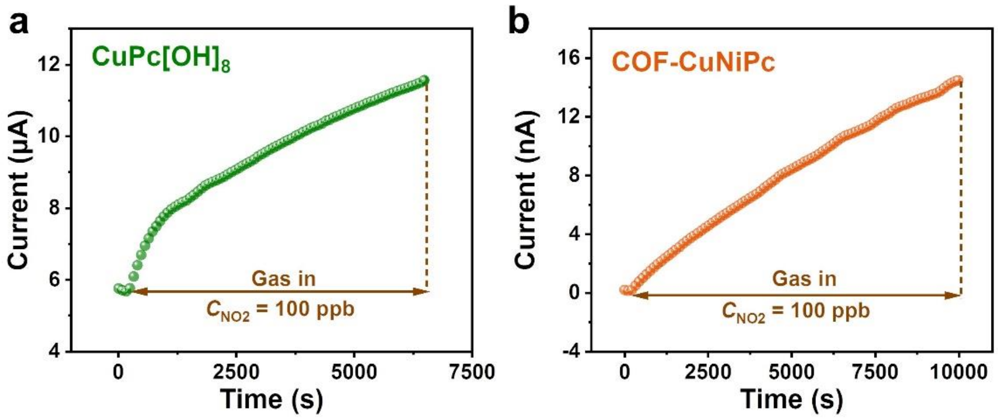

Figure S9. (**a**) Dynamic response characteristic curves of CuPc[OH]8-based device, and (**b**) COF-CuNiPc-based chemiresistive device exposed to 100 ppb NO_2 for a long time (>6000 s) at RT.

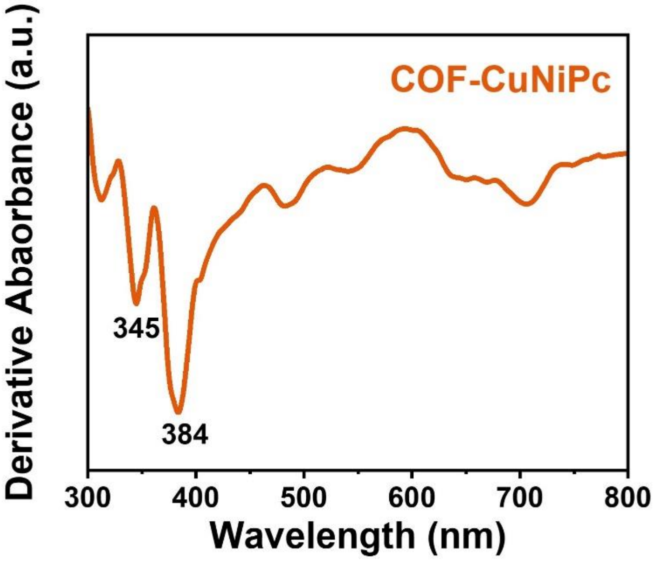

Figure S10. The absorption peak positions of COF-CuNiPc were differentiated once with the assistance of the absorption spectrum of UV-Vis DRS.

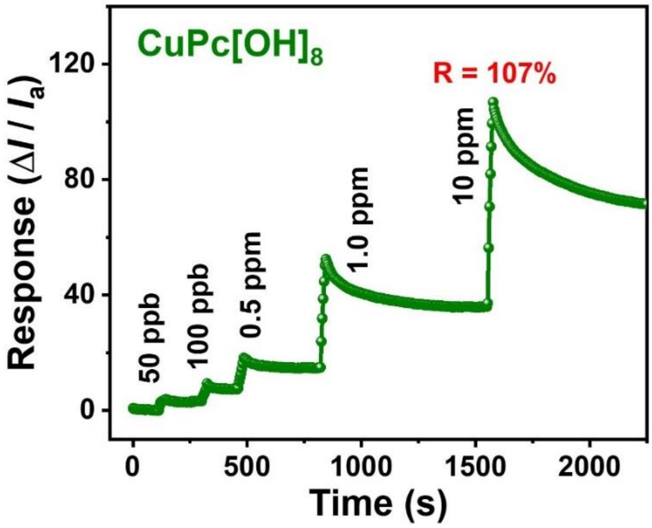

Figure S11. Dynamic current characteristic curve of CuPc[OH]$_8$-based sensor exposed to different concentrations of NO_2 (0.05, 0.1, 0.5, 1.0, and 10 ppm) with UV-assisted recovery.

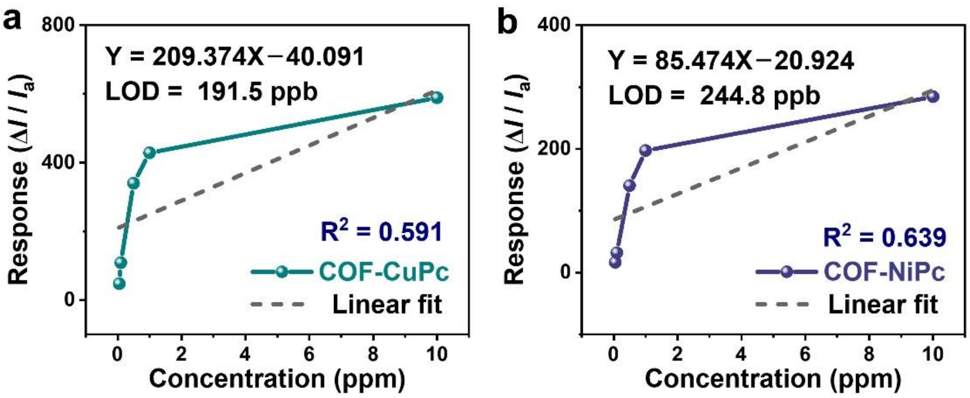

Figure S12. The linear fit curve of response versus the concentration absorption for the gas sensors based on (**a**) COF-CuPc and (**b**) COF-NiPc.

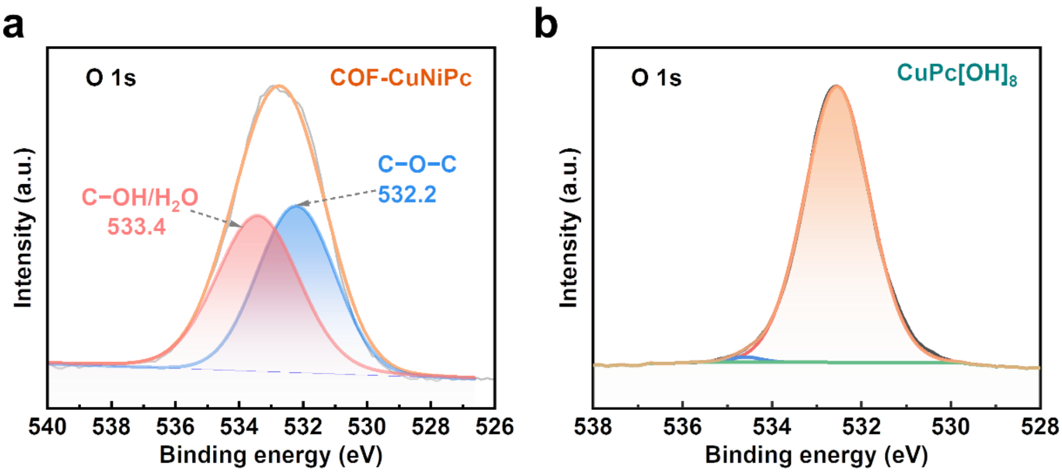

Figure S13. High-resolution O 1s core-level XPS spectra of (**a**) COF-CuNiPc and (**b**) CuPc[OH]8.
